# Directed Self-Assembly of Heterologously Expressed Hagfish EsTKα and EsTKγ for Functional Hydrogel

**DOI:** 10.3389/fbioe.2022.960586

**Published:** 2022-07-22

**Authors:** Ruishuang Sun, Ruonan Zheng, Wenlong Zhu, Xiqin Zhou, Luo Liu, Hui Cao

**Affiliations:** Beijing Bioprocess Key Laboratory, Beijing University of Chemical Technology, Beijing, China

**Keywords:** hagfish slime protein, recombinant protein, self-assembly, phase separation, isolation, purification

## Abstract

Hagfish slime proteins have long been considered useful due to their potential applications in novel green, environmental, and functional bionic materials. The two main component proteins in the slime thread of hagfish, (opt)EsTKα and (opt)EsTKγ, were used as raw materials. However, the methods available to assemble these two proteins are time- and labor-intensive. The conditions affecting protein self-assembly, such as the pH of the assembly buffer, protein concentration, and the protein addition ratio, were the subject of the present research. Through a series of tests, the self-assembly results of a variety of assembly conditions were explored. Finally, a simplified protein self-assembly method was identified that allows for simple, direct assembly of the two proteins directly. This method does not require protein purification. Under the optimal assembly conditions obtained by exploration, a new gel material was synthesized from the hagfish protein through self-assembly of the (opt)EsTKα and (opt)EsTKγ. This assembly method has the benefits of being a simple, time-saving, and efficient. The self-assembled protein gel products were verified by SDS polyacrylamide gel electrophoresis (SDS-PAGE) and contained (opt)EsTKα and (opt)EsTKγ proteins. Scanning electron microscopy (SEM) was used to investigate the self-assembled protein gel after freeze-drying, and it was observed that the self-assembled protein formed a dense, three-dimensional porous network structure, meaning that it had good water retention. Evaluation of the gel with atomic force microscopy (AFM) indicated that the surface of the protein fiber skeleton show the network-like structure and relatively smooth. Characterization by circular dichroism (CD) and Fourier transform infrared spectroscopy (FT-IR) demonstrated that the two proteins were successfully assembled, and that the assembled protein had a secondary structure dominated by α-helices. The rheological properties of the self-assembled products were tested to confirm that they were indeed hydrogel property.

## Introduction

Hagfish, an ancient chordate, is a seabed-dwelling fish with a soft eel-like body (Lim et al., 2006). There are many glands on both sides of the hagfish’s body for storing slime, and when they are under pressure or attacked, a large amount of defensive slime is secreted from the slime glands (Fudge et al., 2005; Lim et al., 2006). The slime secreted by the slime glands of the hagfish is not affected by benthic high pressure or high salt, and it has high water absorption. Hagfish slime could expand to 10000 times its initial volume within a few milliseconds of contact with seawater (Fudge et al., 2005). The slime has many biological functions, including blocking the parotids of potential predators, which allows the hagfish to escape from danger and survive (Fernholm, 1981; Lim et al., 2006; Zintzen et al., 2011). The slime of hagfish is very unusual. It is composed of both mucin and protein-based fibers, and the protein-based fibers give the slime its strength and toughness (Downing et al., 1981; Fernholm, 1981; Koch et al., 1991).

The protein-based fibers of hagfish slime are mainly composed of axially arranged Intermediate filament (IF) proteins (Fudge et al., 2015). IF proteins comprise an important protein superfamily that plays a crucial-role in cell mechanics. And IF proteins are also critical components of the cytoskeleton in most animal cells (Herrmann and Aebi, 2000). The IF proteins found in hagfish is mainly composed of two types of thread keratins (TKα and TKγ) (Fu et al., 2015). In the present study, we analyzed the Eptatretus stouti thread keratins (EsTKα and EsTKγ). The EsTKα and EsTKγ proteins are homologous to vertebrate type Ⅰ and Ⅱ IF keratins, and the molecular weights of EsTKα and EsTKγ are 66.7 and 62.8 kDa, respectively (Fu et al., 2015).

In a 2015 study, Jing Fu et al. used synthetic biology and other methods to heterologously express two kinds of thread keratins, EsTKα and EsTKγ, in *E. coli*. Subsequently, they obtained EsTKα and EsTKγ artificial hagfish protein through artificial synthesis (Fu et al., 2015). After exploring these proteins under various experimental conditions, they determined the conditions for self-assembly of the two protein coils. Their experiments laid the foundation for engineering biomimetic recombinant artificial hagfish thread keratin materials. In 2017, Jing Fu et al. processed a fiber rich in β-sheet through the artificial slime thread of hagfish generated by recombinant protein expression and then used glutaraldehyde to covalently cross-link the processed fiber to obtain an artificial hagfish protein fiber with high hardness and a modulus value of 20 GPa (Fu et al., 2017). Paula E. Oliveira et al. studied unassembled separate EsTKα and EsTKγ proteins. The highest average tensile strength of fibers spun at a 1:1 ratio of the two proteins reached nearly 200 MPa, with an elastic modulus of 5.7 GPa, which can basically represent the highest tensile strength reported for these proteins in the absence of chemical cross-linking (Oliveira et al., 2021).

The recombinant α-helical structure of hagfish protein is soft, which allows it to be formed into soft composite materials. These materials could be used to make safety helmets and other products that could absorb heavy impacts. However, the intensity of the hagfish protein is higher when it forms the recombinant β-sheet structure. The resulting materials may be used to weave bulletproof vests and other products. Both α-helix and β-sheet hagfish protein materials could improve the performance of protective materials without increasing their weight (Johnson et al., 1994; Pinto et al., 2014). In summary, from the perspective of sustainable development, recombinant hagfish protein materials may become green, environmental, and functional biomimetic materials similar to bioderived materials such as spider silk and silkworm silk, and become an excellent alternative to chemically synthesized materials in the future (Aigner et al., 2018; Ma et al., 2018).

Protein self-assembly technology exists at the convergence of polymer chemistry and supramolecular chemistry. During the assembly process, proteins may be cross-linked through hydrophobic interactions, electrostatic interactions, hydrogen bond interactions, disulfide bonds, or van der Waals forces to form spatial network structures (Cheng et al., 2019). Compared with various polyhydroxy polymers, protein gels have structural advantages and better biodegradability. Phase separation of the protein solution could occur after high-speed centrifugation (Boeynaems et al., 2018). After protein self-assembly, the fully mixed an homogenized assembled protein solution is rearranged so that different concentrations occupy different spatial regions, allowing for separation and purification (Boeynaems et al., 2018). A more convenient and efficient way to achieve purification and separation is by gathering proteins through phase transformation. This process could be achieved based on the various specific interactions between EsTKα and EsTKγ. The two crude, purified proteins obtained by simple washing were directly assembled, and the assembled proteins were subsequently isolated from solutions containing unassembled EsTKα, EsTKγ proteins, and miscellaneous proteins by phase separation. Using this process, we separated and purified the assembly proteins. The existing assembly methods for EsTKα and EsTKγ thread keratins are cumbersome, inefficient, and time-intensive. Therefore, a simplified assembly method is needed. In the present study, we were able to directly assemble the two proteins by altering various conditions to establish a simple, quick, and efficient assembly method without the need for protein purification. Through the principle of phase separation, the assembled protein was concentrated and separated using ultrafiltration centrifugation, and the assembled protein product was obtained. Subsequently, the assembled protein products were characterized by various methods. These findings provide a theoretical basis for further research to improve hagfish protein assembly.

## Materials and Methods

### Experimental Materials

We used the pET-22b (+) expression vector, BL21 (DE3) competent cells, and the specific optimized hagfish protein gene, as well as other materials referred to by Yang et al. (2021). The recombinant plasmids were named pET22b-(opt)EsTKα and pET22b-(opt)EsTKγ.

### Acquisition and Crude Purification of (opt)EsTKα and (opt)EsTKγ Proteins

The expression and washing methods for (opt)EsTKα and (opt)EsTKγ have been previously described (Yang et al., 2021). (opt)EsTKα and (opt)EsTKγ proteins were expressed in *E. coli* and then obtained from inclusion bodies. The bacterial pellet was resuspended in lysis buffer (50 mM Tris, 200 mM NaCl, 1 mM PMSF). The bacteria were then lysed by high-pressure homogenization. The resulting liquid was centrifuged at 4°C and 10000 rpm, for 20 min. The precipitate was resuspended and washed in inclusion body washing buffer 1 (100 mM Tris, 5 mM EDTA, 2 M urea, 2% TritonX-100, 5 mM DTT) and then in inclusion body washing buffer 2 (100 mM Tris, 5 mM EDTA, 5 mM DTT). The precipitate was washed twice with each washing buffer, and the supernatant was discarded after each centrifugation (10000 rpm for 15 min at 4°C). Finally, the washed inclusion body protein was dissolved in a high concentration urea solution (8 M urea, 0.02 M NaH_2_PO_4_, 0.5 M NaCl). SDS-PAGE analysis was then performed to verify the molecular weights of the two proteins.

### Self-Assembly of (opt)EsTKα and (opt)EsTKγ Proteins

The self-assembly conditions of (opt)EsTKα and (opt)EsTKγ proteins were designed according to methods known to promote helical folding, coiled-coil formation, and IF assembly of type Ⅰ and type Ⅱ IF *in vitro* (Herrmann et al., 2002). First, the crudely purified (opt)EsTKα and (opt)EsTKγ proteins were diluted to 0.4 mg/ml with high-concentration urea solution (8 M urea, 0.02 M NaH_2_PO_4_, 0.5 M NaCl). Next, equal volumes of the two protein solutions were mixed. Subsequently, a gradient dialysis of urea concentration (8 M→ 4 M→ 2 M→ 0 M) was performed in Tris buffer (urea concentrations of 4 M, 2 M, and 0 M, respectively, all containing 2 mM Tris, 1 mM DTT, and dialysis bag specifications of MD77, 10 kDa) at pH 7.0, 7.5, 8.0, 8.5, and 9.0. The dialysis temperature was 4°C. A total of 72 h of dialysis were performed. Each urea concentration was dialyzed for 24 h and the dialysate was changed every 12 h. Then, high concentration urea solutions of the two proteins with different protein concentrations (0.2, 0.4, 0.6, 0.8 and 1.0 mg/ml) were prepared. The two protein solutions were mixed in equal volumes and concentrations. A self-assembly experiment of gradient dialysis was carried out in Tris buffer (Urea concentrations of 4 M, 2 M and 0 M, respectively, both containing 2 mM Tris and 1 mM DTT, and the specification of dialysis bag were MD77 and 10 kDa) at 4°C, pH 9. Finally, according to different ratios (1:1, 1:2, 2:1) a concentration of 1 mg/ml (opt)EsTKα and (opt)EsTKγ two protein were added to the high concentration urea solution. The self-assembly experiment of gradient dialysis was carried out in Tris buffer (Urea concentrations of 4 M, 2 M and 0 M, respectively, both containing 2 mM Tris and 1 mM DTT, and the specification of the dialysis bag was MD77 and 10 kDa) at 4°C, pH 9. The self-assembled solution after dialysis was ultrafiltered and concentrated (the specifications of the ultrafiltration tube were 1.5 ml, 10 kDa). The self-assembled products of the two proteins were obtained by centrifugation and concentration at 4°C and 6000 rpm. Two proteins, (opt)EsTKα and (opt)EsTKγ, with protein concentrations of 1 mg/ml, were subjected to gradient dialysis for 72 h in the dialysis solution at 4°C, pH 9, respectively, as described above. Ultrafiltration concentration was then carried out as previously described.

### SDS Polyacrylamide Gel Electrophoresis

The protein products obtained after self-assembly were diluted in different folds. The composition of two proteins, (opt)EsTKα and (opt)EsTKγ, after self-assembly was investigated using SDS-PAGE. The loading volume of the protein marker was 10 μL. The self-assembled protein product was diluted with Tris buffer (2 mM Tris, 1 mM DTT) for different multiples, and the loading volume was 10 μl. Equal concentrations of highly purified (opt)EsTKα and (opt)EsTKγ proteins were mixed in equal volumes, and the total loading volume was 10 μl.

### Scanning Electron Microscopy Observation

The (opt)EsTKα proteins, (opt)EsTKγ proteins and the assembled protein products (pH = 9, (opt)EsTKα and (opt)EsTKγ concentrations were 1 mg/ml, the ratio of the two proteins was 1:1, and the next characterized assembly proteins, are also products formed under this condition) were loaded into a centrifuge tube and snap frozen in liquid nitrogen. Snap freezing of proteins in liquid nitrogen was performed to minimize damage to the assembled structure. The snap frozen assembled protein was then freeze-dried using a vacuum freeze-dryer. The microstructure of the freeze-dried assembled protein gel was observed using a SEM (Hitachi SU1510). A small amount of protein gel sample was taken after vacuum freeze-drying, and affixed to the sample bench using a conductive adhesive. After treatment by ion sputtering gold spray, the samples were placed on the SEM platform. The microstructure of the mixed proteins was observed and photographed at a voltage of 5 kV.

### Atomic Force Microscopy Observations

A small amount of the gel-like assembled protein product was dropped onto the surface of a double-polished silicon wafer. Then, the silicon wafer and the sample were freeze-dried together. Observation was performed at room temperature using AFM (Nanoscope Systems). Silicon nitride high frequency vibration probes were used and the sample was scanned using intelligent mode, tapping mode, and phase imaging techniques. Image processing software was used to analyze the image (Nova image processing software attached to AFM) (Chromy et al., 2003; Stine et al., 2011).

### Water Holding Capacity (WHC) Testing

The assembled protein gel’s WHC was measured according to the centrifugation method of Kocher et al. with slight changes (Kocher and Foegeding, 1993). A certain mass of the gel-like assembly protein sample was weighed and transferred to a centrifuge tube with mass of m_0_. The total mass of the sample and centrifuge tube is represented by m_1_. The sample was centrifuged for 15 min at 4°C for 10000 rpm. After centrifugation, the water in the upper layer of the centrifuge tube was removed and the surface of the protein gel was dried with filter paper. The total mass of the sample and centrifuge tube after centrifugation is represented by m_2_. The following formula represents the WHC calculation. Protein gel samples were measured three times in parallel.

$$\mathrm{WHC\%}=\frac{\left(m_{1}-m_{0}\right)-\left(m_{1}-m_{2}\right)}{m_{1}-m_{0}}$$

### Circular Dichroism Testing

The individually dialyzed (opt)EsTKα and (opt)EsTKγ proteins and the assembled protein product were each diluted to a concentration of 1.0 mg/ml in buffer containing 2 mM Tris and 1 mM DTT. Triplicate measurement was obtained in the wavelength range from 190 to 260 nm, using the step size of 1 nm and the bandwidth of 1 nm (JASCO J-1500 circular dichroism spectrometer). Average spectra and smoothing were calculated on the CD spectral data using spectra manager software, and the wavenumber range was selected as 190–260 nm. The data were saved and uploaded to http://dichroweb.cryst.bbk.ac.uk. The website performed the calculations to obtain the relative content of the protein secondary structure of the assembled protein.

### Fourier Transform Infrared Spectroscopy Testing

The secondary structure of the assembled protein sample was analyzed using the KBr compression method. The compression was performed using the assembled protein samples that had been snap-frozen in liquid nitrogen and freeze-dried. The absorbance of the sample was measured in the range of 400–4000 cm^−1^ (Nicolet IS10 Fourier transform infrared spectrometer). The scanning temperature was 25°C, and 64 scans were performed at a resolution of 4 cm^−1^. Each protein sample was tested three times. The infrared spectra from 1600 to 1700 cm^−1^ were baseline corrected, Gaussian deconvoluted, and processed for second derivatives using Peakfit software. They were then subjected to peak splitting fitting.

### Determination of Rheological Properties

Frequency scanning experiments were performed on the samples using the oscillating mode of the rheometer to measure their dynamic viscoelasticity. The specific parameters were as follows: shear strain (γ) was 1%, the range of frequency (f) was 0.1–10 Hz, and the test temperature was 25°C. The changes of storage modulus (G′), loss modulus (G″), and loss factor (tan δ = G′/G′) of the assembly products similar to gel were measured.

## Results and Analysis

### Protein Assembly and SDS-PAGE

**FIGURE 1:**
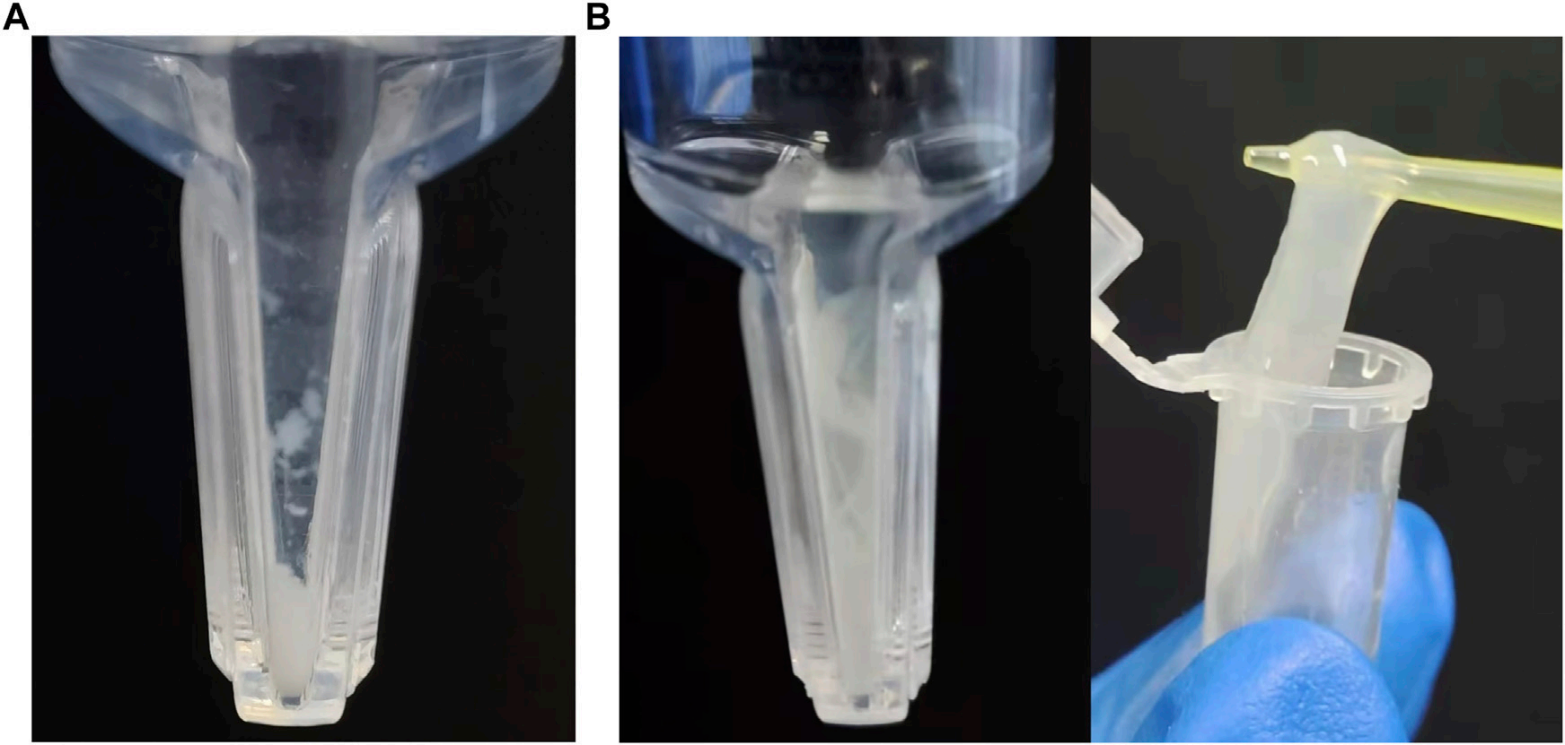
Assembly results at pH 9, 4°C with (opt)EsTKα and (opt)EsTKγ in a 1:1 ratio. **(A)** The concentration of (opt)EsTKα and (opt)EsTKγ were each 0.4 mg/ml. **(B)** The concentration of (opt)EsTKα and (opt)EsTKγ were each 1.0 mg/ml.

In the process of protein assembly, the pH value of the assembly buffer is crucial. Differently pH-ed buffers affect the ionization of proteins and their net charges. This in turn affects the attraction and repulsion between protein molecules, as well as their interactions with water. Our results showed that (opt)EsTKα and (opt)EsTKγ were correctly expressed. When the pH of the protein self-assembly dialysis solution was 7.0, 7.5, 8.0, or 8.5, and the protein solution assembled by dialysis was concentrated by ultrafiltration centrifugation, the inner tube of the ultrafiltration tube showed a clear liquid state. When the pH of the dialysate was 9.0, a small amount of white flocculent substances were visible in the ultrafiltration tube after the assembly protein solution was concentrated by ultrafiltration centrifugation (Figure 1A). Subsequently, the pH of the protein self-assembled dialysate was fixed at 9.0, while the concentrations of (opt)EsTKα and (opt)EsTKγ were changed for the self-assembly experiments. Our findings showed that the concentrations of the two proteins increased during self-assembly, and more white substances were obtained by ultrafiltration concentration by the end of assembly. When the concentration of both (opt)EsTKα and (opt)EsTKγ was 1.0 mg/ml and concentrated by ultrafiltration after assembly, a gel-like assembly protein appeared in the solution in the ultrafiltration tube (Figure 1B). This is a physical-process in which supersaturated component solutions spontaneously separate into a dense phase and dilute phase and stably coexist (Boeynaems et al., 2018). The gel-like assembly protein was slowly picked up with a pipette tip to separate it from the precipitated (opt)EsTKα or (opt)EsTKγ proteins and miscellaneous proteins. This process enables the simple, rapid, and efficient assembly of the two proteins. When performing the assembly of (opt)EsTKα and (opt)EsTKγ proteins in different ratios, it was found that the experimental results after changing the ratio were similar to those when the addition ratio was 1:1. The two proteins, EsTKα and EsTKγ, were purified as described by Jing Fu et al. using size exclusion chromatography (Fu et al., 2015). Both proteins were diluted with a high concentration urea solution to concentrations of 0.2 mg/ml. Self-assembly was successfully performed by adding the two proteins in equal proportions at a dialysate pH of 8.4 and a temperature of 4°C. Because the crude purified protein still contains some miscellaneous proteins that could interfere with the interaction between (opt)EsTKα and (opt)EsTKγ, the use of crude purified protein in this method of direct self-assembly requires increasing the concentration of two proteins, (opt)EsTKα and (opt)EsTKγ, during assembly. This allows them to resist the effects of the miscellaneous proteins so that the two proteins could be successfully assembled.

**FIGURE 2:**
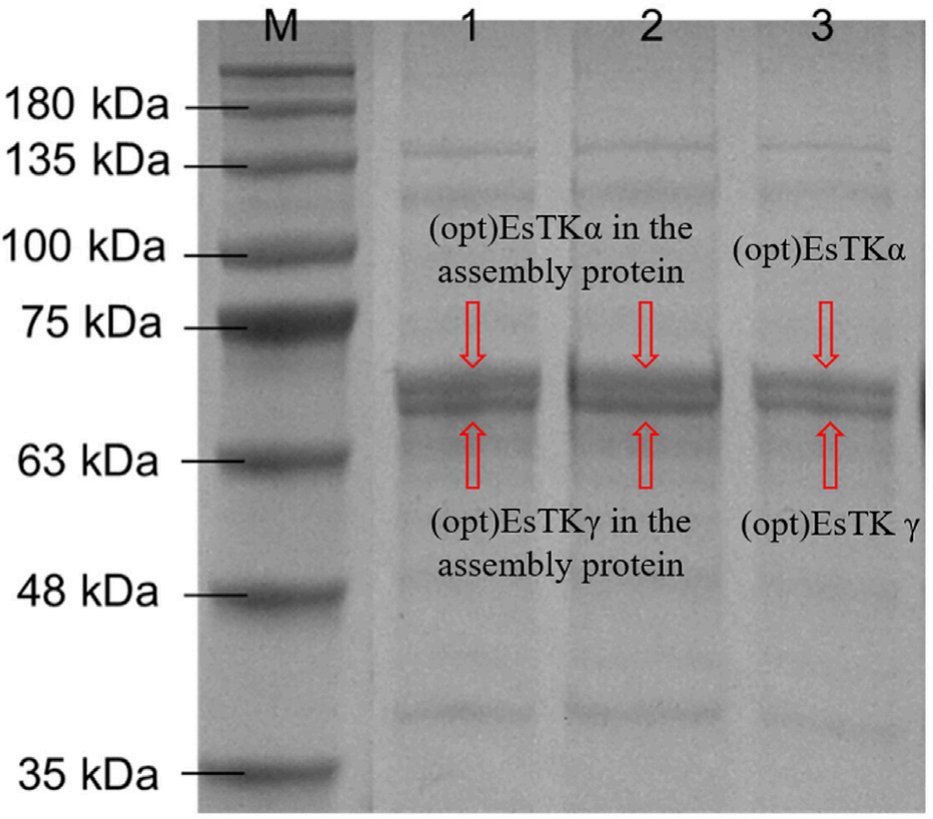
Results of SDS-PAGE. M: Marker. 1, 2: Assembled proteins at different diluted. 3: Direct mixture of (opt)EsTKα and (opt)EsTKγ.

The gel-like assembled proteins were diluted at different concentrations. SDS-PAGE was then performed to validate each component of the gelatinous protein. The process of boiling at high temperature before electrophoresis will denature and disintegrate the assembled gelatinous histones. Using this method, it is possible to induce the formation of depolymers of the two proteins at the target band positions (Figure 2). We observed almost no miscellaneous protein on the SDS-PAGE gel. The electrophoretic bands from the assembled sample corresponded identically to the bands observed from the direct mixture of the two proteins. The molecular weight expressed was consistent with the molecular weights of (opt)EsTKα and (opt)EsTKγ proteins (66.7 and 62.8 kDa, respectively). The bands of the two proteins contained essentially the same amount of protein, indicating that the ratio of the two proteins in the assembled protein was approximately 1:1. We calculated that approximately 4 ml of gel-shaped assembled protein would be formed per 10 mg of a mixed (opt)EsTKα and (opt)EsTKγ (where the ratio of (opt)EsTKα and (opt)EsTKγ was 1:1 and the concentration was 1.0 mg/ml for both).

### Microstructure of Assembled Proteins

**FIGURE 3:**
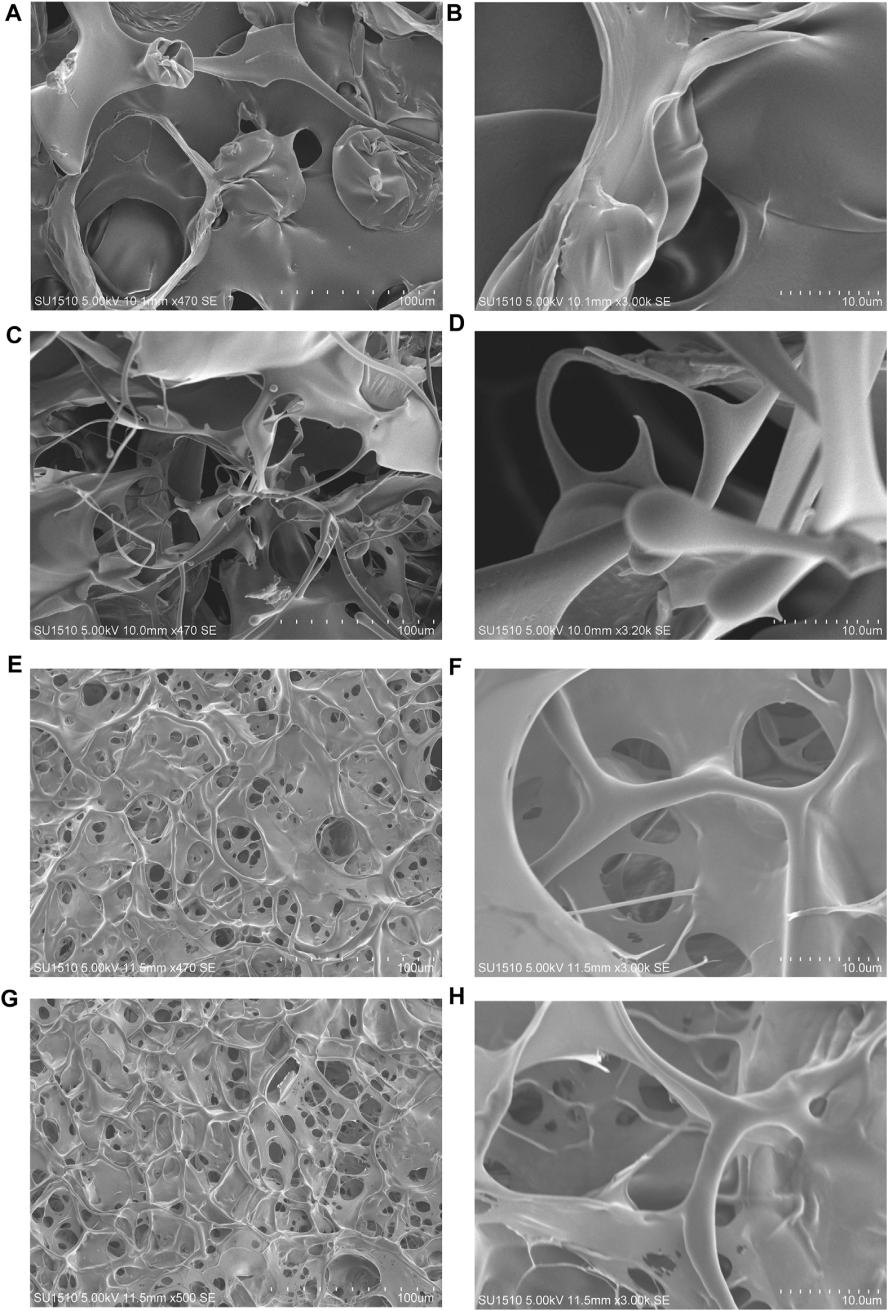
The surface morphology observed by SEM. **(A,B)** The surface morphology of the (opt)EsTKα protein sample. **(C,D)** The surface morphology of the (opt)EsTKγ protein sample. **(E–H)** The surface morphology of the self-assembled protein sample.

The surface morphologies of the (opt)EsTKα and (opt)EsTKγ proteins and the self-assembled protein samples after liquid nitrogen snap freezing and freeze-drying could be clearly observed by scanning electron microscopy. Figures 3A–D shows that the surface microstructure of (opt)EsTKα and (opt)EsTKγ proteins without assembly and treatment was relatively disordered, and there was no mature network structure. Meanwhile Figures 3E–H shows that the proteins of the assembled products self-assembled to form fibrous structures through various interactions between protein molecules. Moreover, these protein fibers formed a clear and dense stereoporous network-like structure through mutual cross-linking and various aggregations. The pores in the network were interlaced and connected with each other, and the pore size was uneven, ranging from 5–25 μm. Amplification of the network skeleton revealed that the mesh skeleton was not uniform in thickness, but the surface was relatively smooth. The diameter of the fibrous network skeleton was in the range of 1–10 μm.

These findings show that using our method of direct assembly, two proteins, (opt)EsTKα and (opt)EsTKγ, could achieve self-assembly at a low temperature. After pure silk fibroin is assembled into a gel, its surface also shows a porous structure, and the size of the pores is also heterogeneous, but its pore size is small, roughly in the range of 2–5 μm (Wu, 2012). Both hagfish slime thread protein and silk fibroin protein are animal-derived fibrins, and their surface morphologies are similar after they are assembled into gels.

**FIGURE 4:**
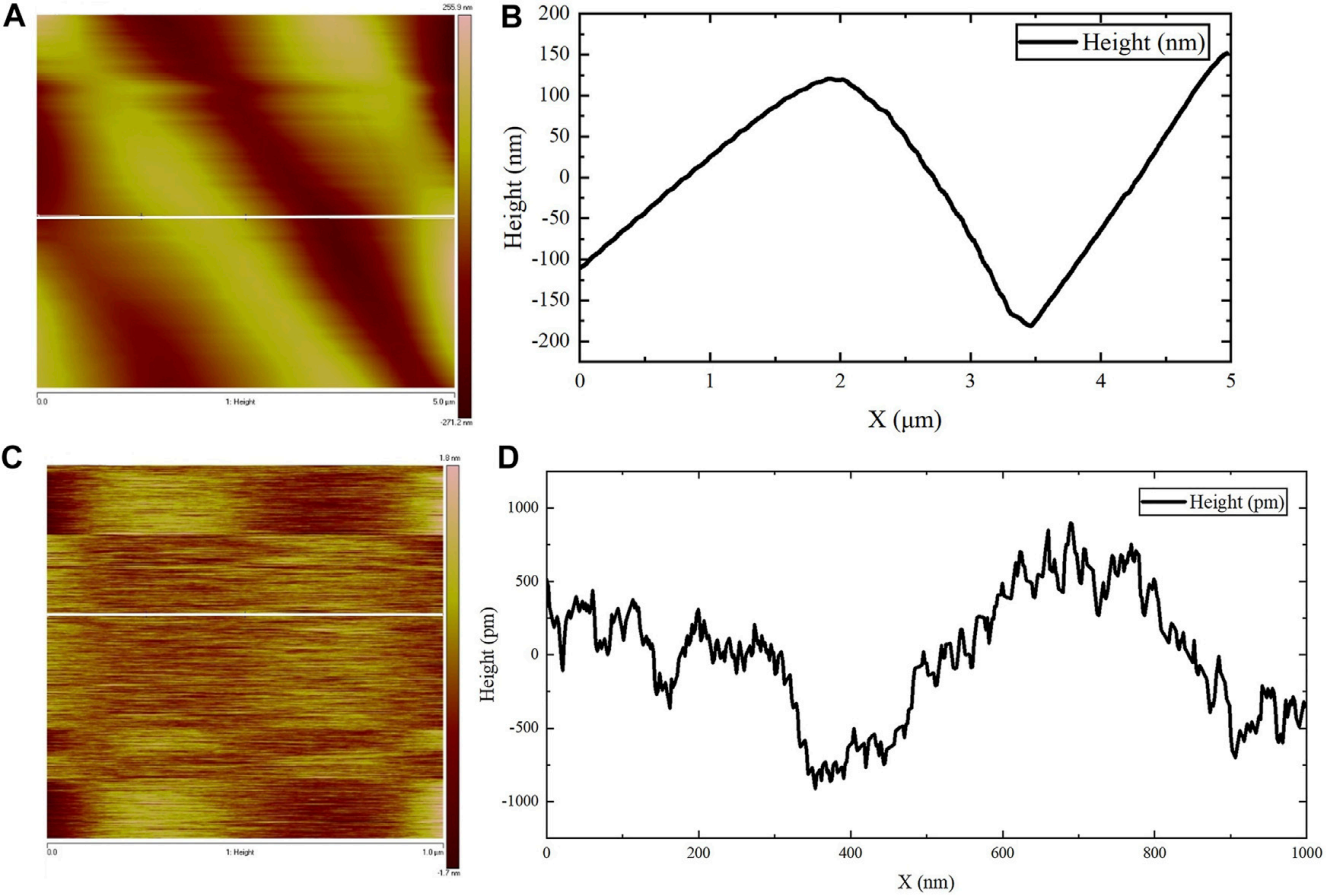
AFM observations. **(A,C)** The surface height map of the protein fiber. **(B)** The surface fluctuation map of the white straight area in **(A)**. **(D)** The surface fluctuation map of the white straight area in **(C)**.

AFM was used to observe the microscopic morphology of the assembled gel protein products at room temperature. Consistent with the previous SEM observation results, the protein gel skeleton was composed of many assembled protein fibers, and the bright and dark staggered stripes formed by the arrangement of the assembled protein fibers were clearly visible (Figure 4A). The surface height could be observed by examining the white, straight part of the protein in Figure 4A, focusing in on the section of the protein mentioned above to further clarify the surface height. Figure 4B represents the surface fluctuation. These images show that the diameter of this protein fiber was about 3.3 µm, which is consistent with the results of SEM observation. Figure 4C shows the amplification of the fiber portion in Figure 4A. The surface height could be observed by examining the white, straight part of the protein in Figure 4C. Figure 4D shows the surface undulation of the protein fiber. Based on these images, the height difference of the undulating surface of the assembled protein fiber was less than 2 nm. The diameter of the protein fiber was thousands of times its surface undulation height, indicating that the assembled protein fiber surface was relatively smooth.

### WHC of Assembled Proteins

Results of the WHC tests are shown in Table 1. Results of the three parallel determinations were close to each other. The average WHC of the assembled protein products was about 66.14%, indicating a good water holding performance. WHC reflects the ability of the gel-like assembled protein network to hold water after freeze-drying, as observed using SEM. SEM indicated that the assembled protein products formed a network-like, three-dimensional structure with relatively dense pores. This structure enables the protein to envelope water and other components, forming a gel. Salvador et al. studied the correlation between gel’s structure and its water holding capacity and found that gels with dense structure were able to hold more water (Salvador et al., 2009). Therefore, the denser porous network structure of the assembled protein product is key to its water holding performance.

**TABLE 1:** Results for WHC.

	m_0_ (mg)	m_1_ (mg)	m_2_ (mg)	WHC (%)
1	860.75	1349.29	1181.09	65.57
2	842.50	1322.24	1173.33	68.96
3	861.29	1352.54	1175.10	63.88

### Secondary Structure of Assembled Proteins

**FIGURE 5:**
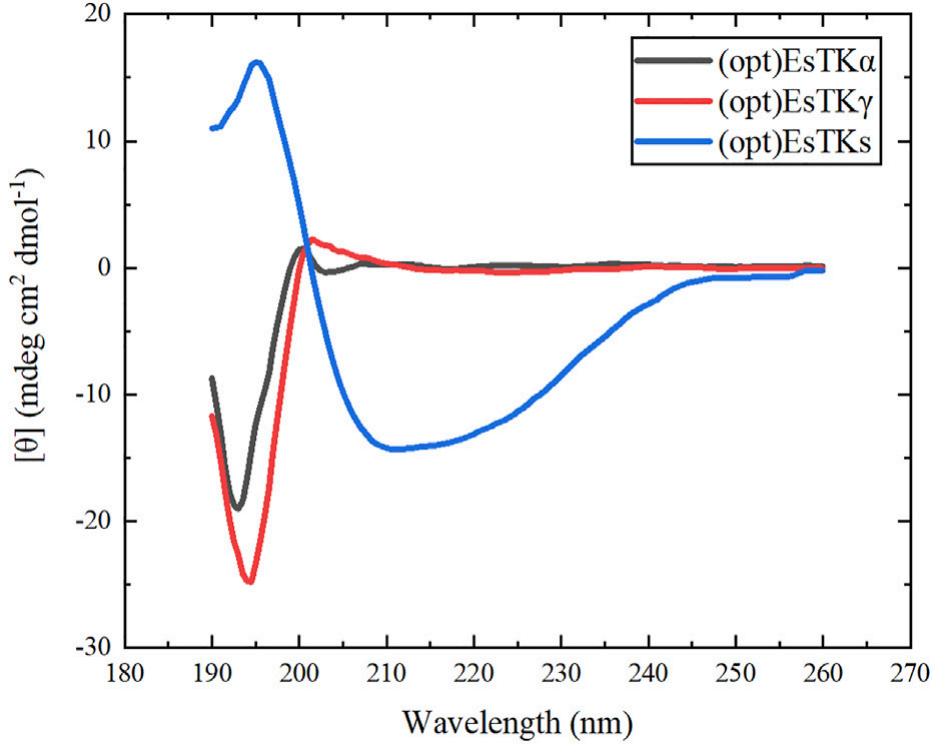
CD spectra of (opt)EsTKα, (opt)EsTKγ and (opt)EsTKs proteins.

CD chromatography accurately reflects the secondary structure of proteins. This method was used to study the secondary structure of the two proteins, (opt)EsTKα and (opt)EsTKγ, and the assembled protein [(opt)EsTKs protein]. The CD test was first performed on the single (opt)EsTKα protein and (opt)EsTKγ protein. The assembled (opt)EsTKs protein was then subjected to CD testing. As shown in Figure 5, the CD spectra of the (opt)EsTKα protein showed a negative peak near 193 nm, and the CD spectra of (opt)EsTKγ protein showed a negative peak near 195 nm. Both of these negative peaks are typical of random coil structures (Li et al., 2008). The CD spectrum of the (opt)EsTKs protein showed a positive peak at near 198 nm, which is characteristic of the β-sheet conformation (Li et al., 2008). The CD spectrum of the assembled (opt)EsTKs protein showed a strong negative peak near 210 nm. The intensity ratio of 222/208 in the CD output of (opt)EsTKs protein was 0.93. Proteins with a 222/208 nm ratio above 1 are considered to exhibit coiled-coil folding (Frère et al., 1995). Data processing was used to calculate the relative content of each secondary structure (α-helix, β-sheet, β-turn, random coil) in the assembled protein product. The assembled product was comprised of 43.0% α-helix, 21.9% β-turn, 11.2% β-sheet, and 23.9% random coil. By comparing the CD test results before and after assembly of the two proteins, we observed that the assembled (opt)EsTKα and (opt)EsTKγ proteins changed dramatically in their secondary structures due to the interactions between the two protein molecules. Specifically, the proteins changed their secondary structures from random coil to α-helix. Jing Fu et al. previously assembled the purified EsTKα and EsTKγ proteins (Fu et al., 2015). Based on their CD analysis, the CD spectra of Jing Fu et al.’s assembled protein products had obvious minimum values at 208 and 222 nm. The 222/208 intensity ratio in the CD results for the assembled proteins was 0.92 (Fu et al., 2015). Jing Fu et al.’s assembled products also consisted primarily of ⍺-helix. Therefore, the assembled proteins obtained by our method and Fu et al.’s method were similar in their secondary structures.

**FIGURE 6:**
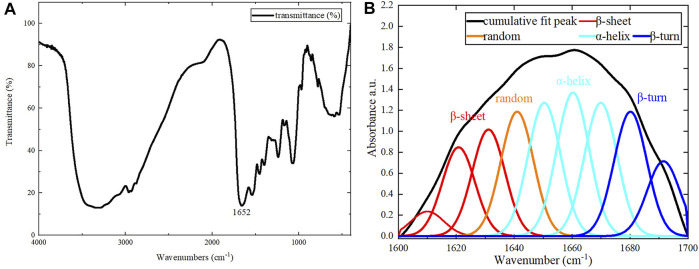
FT-IR results. **(A)** Results for the assembled protein in the range of 400–4000 cm^−1^. **(B)** The peak separation of the amide Ⅰ band.

For the assembled protein products, FT-IR tests were performed in addition to CD tests. The FT-IR spectrum is shown in Figure 6A. The amide Ⅰ band (1600–1700 cm^−1^) in the FT-IR spectrum contains abundant secondary structure information (α-helix, β-sheet, β-turn, random coil, etc.), and was the focus of our analysis. Different sub-peaks were obtained by deconvolution and Gaussian fitting of the amide Ⅰ band (Figure 6B). By analyzing the amide Ⅰ band, the α-helix content was 42.8%, the β-turn content was 20.3%, the β-sheet content was 23.9%, and the random coil content was 13.0% (Wu et al., 2017; Dong et al., 2021). There were some differences between CD and FT-IR analyses of the assembled protein products, due to the limitations of each method. Therefore, the exact secondary structure compositions obtained by CD and FT-IR were not identical. However, both methods were consistent in their determination that the secondary structure of the assembled protein consists primarily of ⍺-helix.

### Rheology of Assembled Proteins

**FIGURE 7:**
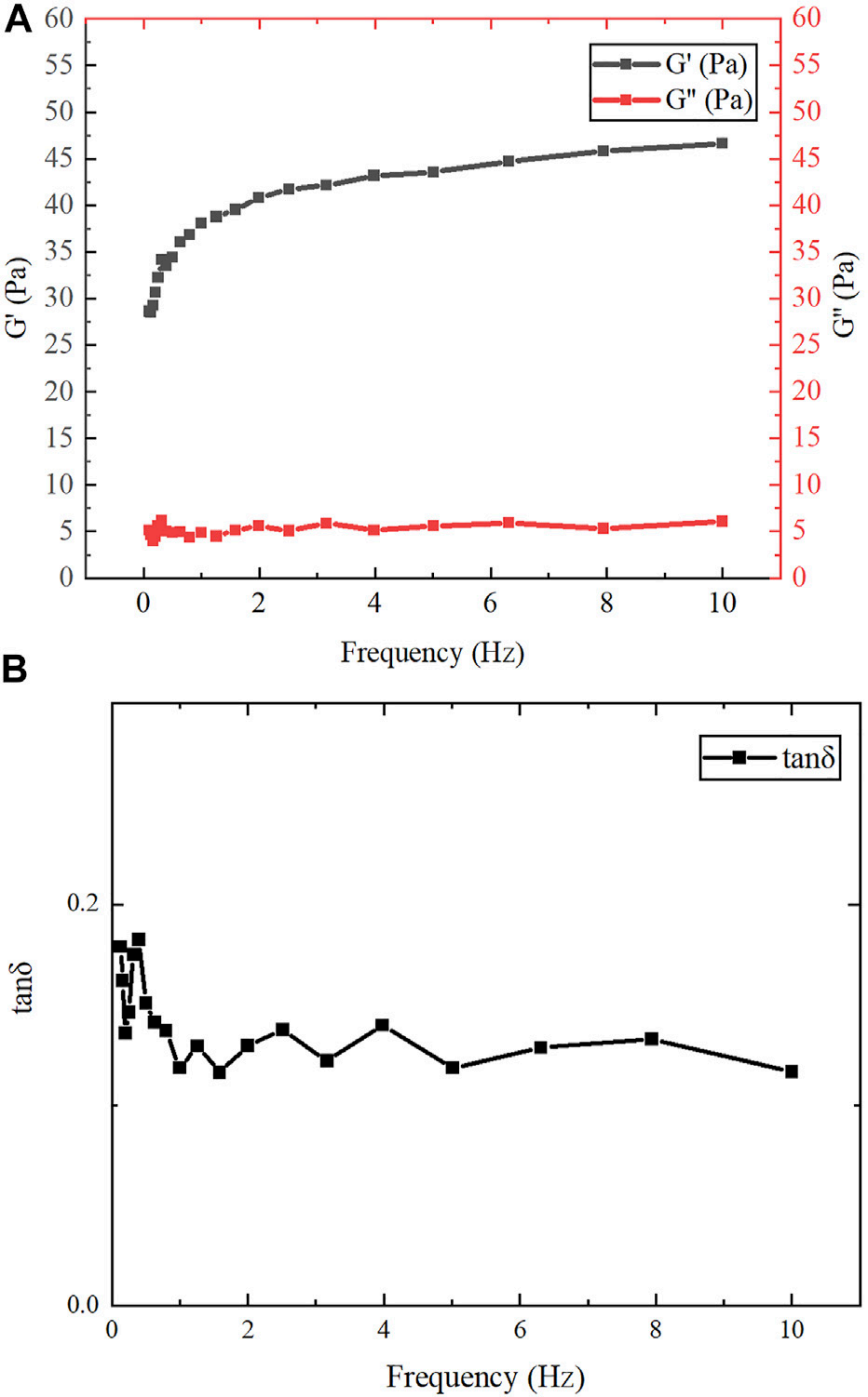
Rheological test results. **(A)** The variation of G′ and G″ of gelatinous products in the range of f (0.1–10 Hz). **(B)** The variation of tan δ in the range of f (0.1–10 Hz).

After assembly at low temperature, the protein product was milky white, transparent, and flowing. The dynamic frequency scanning results of the assembled protein products under the conditions of 1% γ and 0.1–10 Hz f are shown in Figure 7A. In the entire measured frequency range, the change of G″ is not obvious, but G′ gradually increases with the increase of f. Therefore, compared with G″, G′ plays a leading role in the entire f range. This shows that the dependence of the viscoelastic modulus on oscillation frequency is relatively high, which means that the overall chain mobility of the network is relatively high (Lopes-da-Silva et al., 2007). As shown in Figure 7B, the change of tan δ was not obvious in the measured frequency range and was always less than 1, indicating that the assembled protein products showed gel-like behavior (Fu et al., 2015). Jing Fu et al. also found through experiments that all 1:1 mixtures of purified proteins at concentrations higher than 0.2 mg/ml showed gel-like behavior (G″/G '< 1) (Fu et al., 2015).

## Conclusion

The present study demonstrates direct self-assembly of (opt)EsTK⍺ and (opt)EsTKγ under the assembly conditions of 4°C, dialysis solution pH 9, and a 1:1 starting ratio of (opt)EsTK⍺: (opt)EsTKγ both at 1.0 mg/ml. After self-assembly, the two proteins form a new protein gel material. This method of direct assembly omits the need for protein purification, which saves time, allows for rapid and efficient protein assembly. The assembled protein gel was determined by SDS-PAGE analysis to have roughly equal proportions of (opt)EsTKα and (opt)EsTKγ proteins. We calculated that 5 mg each of (opt)EsTK⍺ and (opt)EsTKγ would form approximately 4 ml of gel-like assembled protein. The protein gels after freeze-drying were observed using SEM and AFM. After self-assembly, the protein had many fiber structures with indications of a smooth surface. These protein fibers cross and aggregate to create a three-dimensional porous network structure with dense pores, forming the backbone of the protein gel. This network structure provides the assembled proteins with good water holding capacity. The rheological property test also showed that this new protein material exhibited gel-like behavior. CD analysis of the two proteins before and after assembly showed that the two proteins had random coil structures before self-assembly that changed after self-assembly. These results demonstrate that the method of direct assembly could successfully assemble both proteins. The CD and FT-IR spectra of the assembled protein gels were analyzed to obtain the secondary structure, which was found consist of more than 40% α-helix. The specific interactions between (opt)EsTKα and (opt)EsTKγ and their overall structural characteristics require further study. Due to the excellent biocompatibility and biodegradability of protein-based hydrogel. These properties make it a more widely used potential in the non-food industry field, such as simulating organism tissues, as a carrier for drugs, etc., for biomedical engineering. Moreover, it also has a wide application potential in leather, pharmaceutical, cosmetic, plastic, textile, biochemical and other industries. In addition, protein gels could be applied as water absorbers and retaining agents in a variety of fields due to their water absorption and retainment abilities. However, poor salt tolerance is an insurmountable drawback for most existing hydrogels. The hagfish slime protein used in this study is a potential solution to this challenge because it comes from the ocean and has a unique salt tolerance. In future studies, the hagfish slime assembled protein gel could be complexed with a polyaspartic acid hydrogel to prepare a high-performance biomimetic salt-tolerant hydrogel. It may replace some synthetic substances derived from oil and become a high-performance green and environmentally friendly, sustainable natural material.

## Data Availability

The original contributions presented in the study are included in the article/supplementary material, further inquiries can be directed to the corresponding author.
